# Population Cohort-Validated PM_2.5_-Induced Gene Signatures: A Machine Learning Approach to Individual Exposure Prediction

**DOI:** 10.3390/toxics13070562

**Published:** 2025-06-30

**Authors:** Yu-Chung Wei, Wen-Chi Cheng, Pinpin Lin, Zhi-Yao Zhang, Chi-Hsien Chen, Chih-Da Wu, Yue Leon Guo, Hung-Jung Wang

**Affiliations:** 1 Graduate Institute of Statistics and Information Science, National Changhua University of Education, Changhua City 500207, Taiwan; weiyuchung@cc.ncue.edu.tw; 2 Institute of Medical Sciences, Tzu Chi University, Hualien City 970374, Taiwan; wccheng888@gmail.com; 3 National Institute of Environmental Health Sciences, National Health Research Institutes, Zhunan 35041, Taiwan; pplin@nhri.edu.tw (P.L.); chidawu@ncku.edu.tw (C.-D.W.); leonguo@ntu.edu.tw (Y.L.G.); 4 Institute of Biomedical Sciences and Engineering, Tzu Chi University, Hualien City 970374, Taiwan; 112337103@gms.tcu.edu.tw; 5 Department of Environmental and Occupational Medicine, National Taiwan University College of Medicine and National Taiwan University Hospital, Taipei 100225, Taiwan; chchen66619@gmail.com; 6 Department of Geomatics, College of Engineering, National Cheng Kung University, Tainan 701401, Taiwan; 7 Research Center for Precision Environmental Medicine, Kaohsiung Medical University, Kaohsiung 807378, Taiwan; 8 Innovation and Development Center of Sustainable Agriculture, National Chung Hsing University, Taichung 402202, Taiwan; 9 Institute of Environmental and Occupational Health Sciences, National Taiwan University College of Public Health, Taipei 106319, Taiwan; 10 Doctoral Degree Program in Translational Medicine, Tzu Chi University and Academia Sinica, Hualien 97004, Taiwan

**Keywords:** PM_2.5_, transcriptomic profiling, biomarker, machine learning, predictive model

## Abstract

Transcriptomic profiling has shown that exposure to PM_2.5_, a common air pollutant, can modulate gene expression, which has been linked to negative health effects and diseases. However, there are few population-based cohort studies on the association between PM_2.5_ exposure and specific gene set expression. In this study, we used an unbiased transcriptomic profiling approach to examine gene expression in a mouse model exposed to PM_2.5_ and to identify PM_2.5_-responsive genes. The gene expressions were further validated in both the human cell lines and a population-based cohort study. Two cohorts of healthy older adults (aged ≥ 65 years) were recruited from regions characterized by differing levels of PM_2.5_. Logistic regression and decision tree algorithms were then utilized to construct predictive models for PM_2.5_ exposure based on these gene expression profiles. Our results indicated that the expression of five genes (*FAM102B*, *PPP2R1B*, *OXR1*, *ITGAM*, and *PRP38B)* increased with PM_2.5_ exposure in both cell-based assay and population-based cohort studies. Furthermore, the predictive models demonstrated high accuracy in classifying high-and-low PM_2.5_ exposure, potentially supporting the integration of gene biomarkers into public health practices.

## 1. Introduction

Exposure to air pollutants, especially in the form of fine particulate matter (PM_2.5_), represents a significant threat to human health. PM_2.5_ can deeply penetrate and infiltrate the pulmonary system, even entering the circulatory system and blood–brain barrier into the central nerve system, leading to various adverse health effects. Numerous studies have linked PM_2.5_ exposure to elevated risks of severe conditions, including heart disease, lung cancer, respiratory diseases, stroke, diabetes, and pregnancy complications [1,2,3,4,5]. According to the World Health Organization, long-term PM_2.5_ exposure contributed to 4.2 million premature deaths worldwide in 2019, accounting for 62% of all air pollution-related deaths [6]. Therefore, mitigating the adverse health impacts linked to PM_2.5_ is an essential public health concern, requiring multifaceted strategies for environmental intervention. Such strategies may include monitoring air quality to identify high-risk regions, implementing policies to reduce emissions from industrial and transportation sources, and enhancing public awareness about the health consequences of air pollution.

Considering the intricate composition of PM_2.5_, which comprises a variety of metals, organic compounds, microorganisms, and other hazardous chemical constituents, it can be hypothesized that exposure to PM_2.5_ may have broader implications that extend beyond respiratory and cardiovascular health. Numerous studies have established associations between PM_2.5_ exposure and the development of chronic diseases, such as neurodegenerative disorders and immune system dysfunctions [7,8,9]. These results highlight the urgent needs for more comprehensive investigations into how PM_2.5_ impacts human health and the cellular pathways mediating these effects. In light of this, developing PM_2.5_ exposure-specific biomarkers that can help assess exposure levels, reveal biological impacts, and forecast health outcomes has generated growing interest. With the accumulated evidences, PM_2.5_ exposure is primarily linked to the generation of reactive oxygen species (ROS) and the induction of numerous oxidative stress-dependent pathways, including inflammatory diseases [10,11]. Although the ROS generated by PM_2.5_ exposure are linked to increased local and systemic inflammation, such as elevated interleukin (IL)-1 β and IL-6 levels [12], using these proinflammatory markers alone lacks specificity for accurately reflecting PM_2.5_ exposure, as many other factors can also trigger similar immune responses.

The advancement of omics-based approaches, such as transcriptomics, proteomics, and metabolomics, has significantly enhanced the investigation of potential biomarkers for PM_2.5_ exposure. These approaches facilitate the identification of PM_2.5_-associated cellular pathways connecting to the various biological outcomes and health implications. For example, exposure to PM_2.5_ has been shown to alter the expression of numerous genes involved in inflammatory and immune responses. This occurs particularly through the activation of key signaling mechanisms, such as MAPK and NF-κB pathways, which upregulate proinflammatory cytokines, exacerbate inflammation, and further damage the lungs and tissues [13,14,15]. Transcriptomic profiling also revealed that PM_2.5_ increases the expression of ROS-related genes in alveolar macrophages, cardiomyocytes, and other cell types, disrupting antioxidant defense mechanisms and damaging cellular components like DNA, proteins, and lipids [16,17,18,19,20]. PM_2.5_ exposure has also been implicated in promoting cancer initiation and progression through several mechanisms including miRNA downregulation, oncogene activation, and Tumor Microenvironment Modulation [21,22]. Collectively, these transcriptomic studies highlight the complex, diverse, heterogeneous, and multi-targeted effects of PM_2.5_ on health burden, reflecting the high dependence on the composition of PM_2.5_ and vulnerability to the exposure.

Although omics-based screening methods having provided comprehensive approaches for identifying potential biomarkers for PM_2.5_ exposure, further evaluation and validation through population-based cohort studies are needed to translate these markers into measurable and reliable early warning indicators for assessing individual exposure to PM_2.5_. Currently, such studies are still limited. In this study, we utilized a PM_2.5_ exposure mouse model followed by transcriptomic profiling with RNA sequencing (RNA-seq) to investigate the cellular pathways linked to PM_2.5_ exposure and identify potential gene expression biomarkers for PM_2.5_ exposure monitoring. We found that the mice exposed to PM_2.5_ exhibited significant effects in their pulmonary and cardiovascular functions, which are consistent with the findings of previous studies [23]. Furthermore, multiple cellular pathways including inflammatory responses and cancer-related signaling cascades were also highlighted in our RNA-seq analysis. The top-ranking genes were further validated by the human cell line-based assay. Among the top-ranking genes, five were further investigated with a population-based cohort study comparing two distinct regions of Taiwan with contrasting PM_2.5_ exposure levels. Our data revealed that all five genes exhibited elevated levels of gene expression in the high PM_2.5_ exposure cohort. Subsequently, we constructed a predictive model to assess PM_2.5_ exposure by employing the cohort study and machine learning algorithms. This model can estimate personal PM_2.5_ exposure based on individual gene expression profiles, potentially transforming environmental health monitoring by providing personalized exposure assessments that account for genetic susceptibility and individual variations in response to PM_2.5_.

## 2. Materials and Methods

### 2.1. PM_2.5_ Sample Collection and Extraction

**Figure 1:**
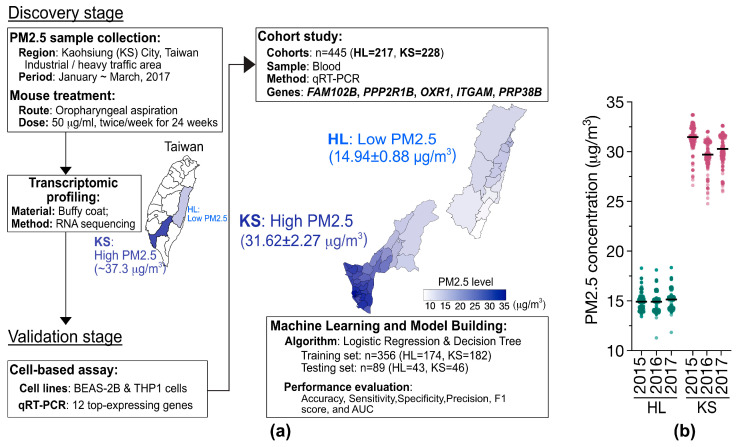
(**a**) Flowchart of a two-stage approach for identifying PM_2.5_-specific gene signatures. In the discovery stage, PM_2.5_ samples from Kaohsiung (KS) City of Taiwan were subjected to mouse studies followed by transcriptomic profiling (RNA-Seq). The average concentration of PM_2.5_ in KS city is indicated. In the validation stage, the candidate genes (12 genes) were verified through cell line-based assays, and the selected 5 genes were further validated using population cohort studies. These validated genes were then utilized to develop predictive models using machine learning approaches. (**b**) Estimated local PM_2.5_ concentrations using an ensemble mixed spatial model approach [25]. The PM_2.5_ exposures of the participants residing in the HL (n = 217) and KS (n = 228) areas of Taiwan from 2015–2018.

PM_2.5_ samples used in this study were collected in Kaohsiung City, Taiwan, during the period from January to March 2017. The average ambient PM_2.5_ concentration in the study area was 37.3 μg/m^3^ (Figure 1a) [23]. The PM_2.5_ samples were collected with a high-volume aerosol sampler DHA-80 (Digitel, Hegnau, Switzerland) operating at a flow rate of 500 L/min [23]. The collected samples were gathered on pre-weighed fiberglass filters (Pallflex Fiberfilm TX40HI20; Pall Corporation, Port Washington, NY, USA). The fiberglass filters containing PM_2.5_ samples were weighed, then moistened with a 70% ethanol solution in a glass beaker, followed by 30 min of ultrasonic agitation at room temperature to extract the PM_2.5_ samples. The ethanol was subsequently removed using an evaporator. The dried PM_2.5_ samples were stored in a pre-weighed, sterile polypropylene container and maintained in a desiccator at room temperature in the dark. The measurement of the weights of the PM_2.5_ samples, fiberglass filters, and polypropylene containers were carried out using a microbalance (AG204, Dual Range; Mettler Toledo, Columbus, OH, USA) in an environmentally controlled room (temperature: 23 ± 1 °C; relative humidity: 40 ± 5%).

### 2.2. PM_2.5_ Exposure Mouse Experiments

The mouse study was conducted at Taiwan’s National Health Research Institutes (NHRI) as described previously [23]. The Institutional Animal Care and Use Committee of NHRI approved the study protocol. In brief, eight-week-old male B6.D2N mice were housed under a 12 h light/dark cycle, at a temperature of 23 ± 1 °C and a relative humidity of 39–43%. Food (Oriental Yeast Co., MFG, Tokyo, Japan) and water were provided to the mice ad libitum. Twenty mice were randomly divided into two groups (10 mice for each group). The experimental group received 25 μg of PM_2.5_ samples via oropharyngeal aspiration twice weekly and were euthanized 24 weeks after the initial PM_2.5_ exposure. The control group received distilled water via the same route of administration. Throughout the study, the mice’s body weights, heart rates, and blood pressures were monitored weekly. The urine and blood samples were collected prior to sacrifice. The internal organs, including the lungs and pancreas, were harvested to determine the organ weights and conduct immunohistochemical analysis. The animal experiment was conducted with protocols by IACUC (Institutional Animal Care and Use Committee) laboratory animal center of NHRI (NHRI-IACUC-107001-A).

### 2.3. IHC Analysis

The pulmonary tissues of mice were fixed with a 4% formaldehyde solution for 24 h, followed by alcohol dehydration, paraffin embedding, and sectioning (5 µm). Angiogenesis and inflammatory cell infiltration in the lungs were evaluated by using H&E (hematoxylin and eosin) staining. S100A4 expression in the tissues was examined using IHC (immunohistochemistry) with a specific antibody to S1004A (GeneTex, Irvine, CA, USA, Cat. No. 32855), and hematoxylin was used for nuclear staining. The stained slides were examined under a 200× optical microscope.

### 2.4. Transcriptomic Profiling of PM_2.5_-Treated Mice

#### 2.4.1. Buffy Coat Isolation and RNA Extraction

Whole blood was collected in PAXgene^®^ Blood RNA Tubes (BD Biosciences, Cat. No. 762165) for the purpose of buffy coat isolation. Equivalent volumes of EasyPre buffer (1X PBS containing 2% of Fetal bovine serum, and 1 mM EDTA) (STEMCELL Technologies, Vancouver, Canada, Cat. No. 20144) were combined and agitated. The mixtures were centrifuged at 800× *g* for 10 min with break off at room temperature. The buffy coat samples were collected and dissolved in TRIzol Reagent (Thermo Fisher Scientific, Waltham, MA, USA, Cat. No. 15596018) and followed by total RNA extraction according to the manufacturer’s instructions. Total RNAs were further purified using the RNeasy Mini Kit (Qiagen N.V., Venlo, Netherlands; Hilden, Germany, Cat. No. 74104) following the manufacturers’ instructions.

#### 2.4.2. RNA-Seq Analysis

The purified RNA samples were analyzed using the Agilent 2100 Bioanalyzer (Agilent Technologies, Santa Clara, CA, USA) to assess their quality. Their concentrations were then determined with NanoDrop (ThermoFisher Scientific, Waltham, MA, USA). A total of 1 μg of total RNA with a RIN (RNA integrity number) value above 7 was used for the subsequent library preparation. The library for next-generation sequencing was constructed in accordance with the manufacturer’s protocol of the TruSeqTM RNA sample preparation Kit (Illumina, San Diego, CA, USA). The library size was selected on 2% LowRange Ultra Agarose (ThermoFisher Scientific, Waltham, MA, USA) and then amplified using Phusion DNA polymerase (ThermoFisher Scientific, Waltham, MA, USA) in a 15-cycle PCR procedure. Paired-end sequencing was performed with the Illumina HiSeq XTM Ten by the Next-Generation Sequencing company (BIOTOOLS Co., Ltd., New Taipei City, Taiwan). The raw paired-end reads were trimmed and quality-controlled using Cutadapt (v1.9.1) and Trimmomatic (v0.30) with default parameters. The clean reads were then separately aligned to the reference genome (Mus_musculus_Ensembl_GRCm38.97) using the orientation mode feature of the Hisat2 (v2.0.1) software. To identify the DEGs (differential expression genes) between two different groups, the expression level of each transcript was calculated according to the fragments per kilobase of exon per million mapped reads (FRKM) method by using RSEM (v1.2.6). The PCA (principal component analysis) plot for the two groups was generated using the “prcomp()” function from the “stats” package in R. The log10 (FPKM + 1) values are used for clustering analysis and represented as a heatmap. Gene set enrichment analysis was conducted using GESA 3.0 according to the manual’s instructions. A matrix showing gene expression in each group was used as the input data. Volcano plot was utilized to choose the differentially expressed genes’ DEGs in the transcriptomic analysis. The top 10 representative gene sets selected for each subtype (*p* < 0.05) were used for the visualization heatmap of DEGs using “pheatmap“ R package. The gene set gmt files for GO, KEGG, and HALLMARK were obtained from the MSigDB (Molecular Signatures Database) located at http://www.gsea-msigdb.org. Enrichment analysis was performed utilizing the “clusterProfiler“ R package.

### 2.5. Cell Line-Based Analysis

Two human cell lines, BEAS-2B (bronchial epithelial cells, ATCC CRL-9609) and TPH-1 (monocyte cells, ATCC TIB-202) used to investigate the impact of PM_2.5_ exposure on gene expression were obtained from the American Type Culture Collection (Manassas, VA, USA). The cells were cultured in Dulbecco’s modified Eagle’s medium (Gibco, Carlsbad, CA, USA) supplemented with 4 mM L-glutamine, 1.5 g/L sodium bicarbonate, and 10% heat-inactivated fetal bovine serum. The cells were maintained at 37 °C in a saturated, humidified atmosphere with 5% CO_2_ and 95% air. For the experiments, the cells were exposed to PM_2.5_ samples prepared as previously mentioned at various concentrations (25 ug/mL and 50 ug/mL) and for different time periods (24 h and 48 h). Following the treatments, the cells were washed twice with 1X phosphate-buffered saline and then lysed in TRIzol Reagent (ThermoFisher Scientific) for RNA extraction and qRT-PCR analysis.

### 2.6. qRT-PCR and Gene Expression Analysis

Total RNA samples were reverse-transcribed using iScript™ Reverse Transcription Supermix (Bio-Rad, Cat. No. 1708840) to synthesize cDNAs. The expression levels of genes were quantified using qPCR with GoTaq^®^ qPCR Master Mix (Promega, Cat. No. A6001) on a CFX96 real-time PCR detection system (Bio-Rad Laboratories, Hercules, CA, USA). Primer sets for the qPCR analyses are provided in Table S1. The gene expression value Q used to cohort study in this study is defined as 10^6^ × 2^−ΔCt^, where ΔCt = Ct (gene) − Ct (18S rRNA).

### 2.7. Statistical Analysis

Statistical comparisons between the experimental groups and their respective control groups were conducted using the two-tailed Student’s *t*-test with the GraphPad Prism software (Version 10.0). The results are presented as means ± standard deviation. The error bars denote the standard deviation. Differences at the level of *p* < 0.05 were considered significant in comparison. All experiments were independently performed at least three times. Cells and animals were randomly assigned to the experimental groups, and the investigators were blinded to the allocation during the experiments and outcome assessment. RNA-sequencing analysis was conducted using R software (version 4.4.3) as described in the section of RNA-seq analysis.

### 2.8. Cohort Study

The cohort study was conducted between 2016 and 2018, involving 445 healthy elderly participants (age ≥ 65 years old) residing in Taiwan [24]. The participants were recruited through the annual health examination programs for senior citizens hosted by hospitals in Taiwan. To maximize differentiation in PM_2.5_ exposure levels, we selected two hospitals situated in distinct districts of Taiwan with varying ambient PM_2.5_ concentrations. The study sites include Hualien (HL) City (Hualien Tzu-Chi Hospital, Hualien City), which is located in Eastern Taiwan and exhibits low-level PM_2.5_ exposure, and Kaohsiung (KS) City (Kaohsiung Municipal Siaogang Hospital, Kaohsiung City), situated in southwestern Taiwan and characterized by high-level PM_2.5_ exposure (Figure 1b). To minimize potential confounding factors in the cohort study, participants were care-fully selected from a population of healthy, older adults without apparent health conditions, such as cardiological problems, hypertension, or diabetes. Furthermore, to mitigate the potential confounding effects, randomization of the participants was carried out. The cDNA samples from all participants were the residual specimens previously reported in our earlier study [24]. The annual average PM_2.5_ levels in the two regions from 2015 to 2018 were estimated by integrating environmental monitoring data on PM_2.5_ collected by the Taiwan Environmental Protection Agency and an ensemble mixed spatial model [25]. The institutional review boards of all participating institutions approved the study protocol and all of the participants provided written informed consent.

### 2.9. Machine Learning and Predictive Model Building

The expression levels (represented as Q) of the 5 genetic markers were quantified by qPCR for the participants in the cohort study. Box plots generated by GraphPad Prism software were used to visualize the distribution of log_2_(Q) values for the 5 genes in the HL (low PM_2.5_ exposure) and KS (high PM_2.5_ exposure) cohorts. The Q values for the markers in each cohort were summarized by medians and interquartile ranges (IQRs). The Mann–Whitney U Test was performed to compare the medians between the two populations, with statistical significance set at *p* < 0.05. To predict whether a sample belonged to the HL or KS group based on the Q values of the genes, we utilized logistic regression, a conventional method in medical statistics. Additionally, we employed a decision tree algorithm, valued in the field of machine learning for its interpretability. This algorithm not only predicts whether a sample is from Kaohsiung or Hualien but also reveals key markers and their corresponding Q value thresholds, providing useful insights for clinical application.

In the logistic regression analysis, univariate models were initially constructed for each individual marker to evaluate its predictive performance in distinguishing between high exposure and low exposure. Subsequently, a multivariate analysis was conducted, with all 5 markers included as candidate variables in the logistic regression model. A bidirectional stepwise procedure based on the Akaike Information Criterion (AIC) was used to select the optimal markers for inclusion.

For the decision tree analysis, we utilized the Classification and Regression Trees (CART) method [26] with recursive partitioning. Each node in the tree was split based on selected markers and Q-value thresholds to maximize the reduction in Gini impurity. To avoid overfitting, the one standard error rule was applied, resulting in the most parsimonious model by trimming the least important splits.

For model validation and performance comparison, all the samples were randomly divided into training and testing sets, following the commonly used 80%:20% split ratio. The performance of the models was assessed using five standard metrics: accuracy, sensitivity, specificity, precision, and F1 score, evaluated on both the training and testing sets. Additionally, the area under the receiver operating characteristic (ROC) curve, abbreviated as AUC, was calculated for the training set. Finally, using the complete dataset, odds ratios (ORs) with 95% confidence intervals (CIs) for Kaohsiung (high exposure) versus Hualien (low exposure) were estimated using both univariate logistic regression and the optimal multivariate logistic regression model. The final decision tree model was also constructed. All analyses were performed using R software (version 4.4.3). The R packages “corrplot“, “caret“, “rpart“, and “pROC” were used to calculate correlation coefficients, generate heatmaps, split the data into training and testing sets, construct the CART decision tree, and compute the AUC.

### 2.10. Ethics Approval and Consent to Participate

The study was approved by the research ethics committee of National Health Research Institutes (EC1040508-E-R5) and Hualien Tzu-Chi Hospital (IRB110-271-A). All participants signed an informed consent form.

### 2.11. The Use of Generative Artificial Intelligence (GenAI)

A GenAI tool, ChatGPT-4o by OpenAI, was used to assist the authors in creating individual elements of the Graphical Abstract, which were then combined to form the complete Graphical Abstract.

## 3. Results

### 3.1. PM_2.5_ Exposure Mouse Model

With the purpose of identifying practical markers to monitor and mitigate the exposure risks to PM_2.5_, we conducted transcriptomic profiling using RNA sequencing (RNA-Seq) of mice to identify gene sets specifically linked to PM_2.5_ exposure. The investigation design depicted in Figure 1a comprised two stages: the discovery stage and the validation stage. Initially, PM_2.5_ samples were collected from Kaohsiung (KS) City, a region in southwestern Taiwan characterized by significant industrial and petrochemical activities, during the winter of 2017. The PM_2.5_ level was estimated to be a monthly average of 37.3 μg/m^3^ at that time [23,25]. The PM_2.5_ samples were further analyzed by mass spectrometry to determine their chemical composition (Table S2). Our analysis showed that the PM_2.5_ fraction, in addition to inorganic salts and metals, comprises a considerable proportion of organic compounds, including polycyclic aromatic hydrocarbons such as benzo[ghi]perylene, benzo[b]fluoranthene, and benzo[a]pyrenes.

**Figure 2:**
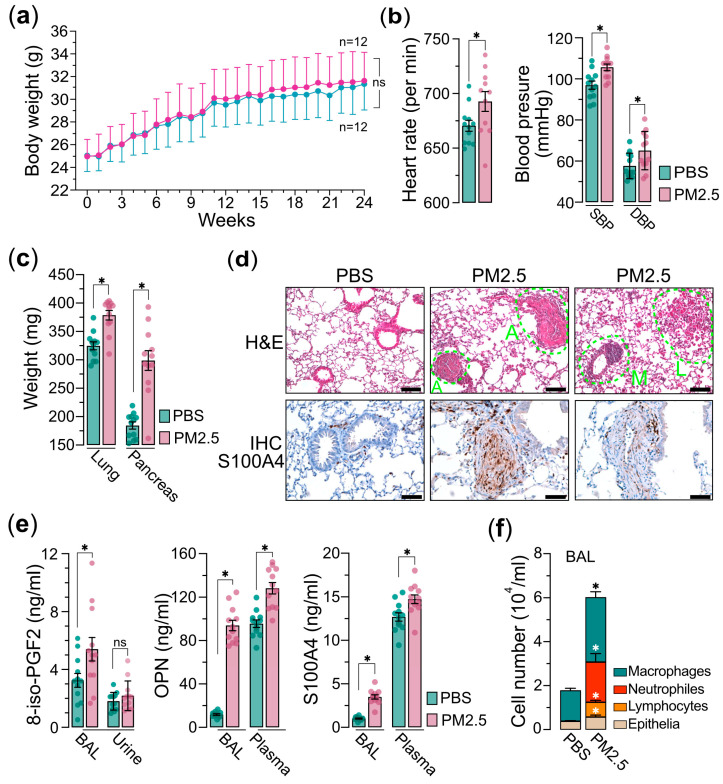
PM_2.5_ exposure mouse model experiments. Mice (n = 24) were administered 25 μg of PM_2.5_ samples (n = 12) or physiological saline (n = 12) via oropharyngeal aspiration twice per week and were euthanized 24 weeks after the initial PM_2.5_ treatment. (**a**) Measurements of mice’s body weights. (**b**) Measurements of mice’s heart rate (**left**) and blood pressure (SBP and DBP are indicated) (**right**) prior to euthanasia. (**c**) Measurements of the organs of lungs and pancreases as indicated. (**d**) Hematoxylin and eosin (H&E) and immunohistochemistry (IHC) analyses of lung tissues of mice. In H&E, A indicates angiogenesis; L indicates leukocyte infiltration; M indicates macrophage activation. In IHC, the lung tissues are marked with specific antibody anti-S100A4. (**e**) Measurements of 8-iso-PGF2 (**left**), OPN (**middle**), and S100A4 (**right**) in bronchoalveolar lavage (BAL), urine, or plasma, as indicated. (**f)** Cell composition in BAL. Immune cells and epithelia cells are indicated. PBS, PBS-treated control mice; PM_2.5_, PM_2.5_-treated mice. Data are presented as the mean ± SEM from independent experiments. Unpaired two-tailed Student’s *t*-tests were performed, with statistical significance defined as *p* < 0.05 (*); ns, not significant.

Next, we administered the PM_2.5_ samples to the mice via the nasal–pharyngeal route twice a week for a duration of 24 weeks. Throughout the exposure period, the body weights of the mice were measured weekly, and we observed a slight, statistically non-significant increase in body weight in the PM_2.5_ exposure group compared to the saline control group (Figure 2a). Prior to euthanasia, two physiological parameters, heart rate and blood pressures, including SBP (systolic blood pressure) and DBP (diastolic blood pressure), were evaluated. We found that the mice exposed to PM_2.5_ exhibited significantly elevated levels of heart rate and blood pressure relative to the control group (Figure 2b). This suggests that PM_2.5_ exposure may trigger systemic oxidative stress and inflammatory processes, potentially resulting in damage to vascular endothelial cells and the advancement of atherosclerosis, which in turn contributes to the observed increases in blood pressure and heart rates [27,28,29,30]. After euthanasia, the weights of internal organs, such as the lungs and pancreas, were measured. Our results revealed that although there were only minor variations in body weight, the weights of the internal organs were significantly higher in the mice exposed to PM_2.5_ compared to the control group (Figure 2c). The result also indicates that exposure to PM_2.5_ has the potential to induce both acute and chronic inflammatory responses within the internal organs. These inflammatory processes may provoke systemic inflammation and metabolic disturbances, which could in turn contribute to the development of several pathological conditions such as insulin resistance and obesity-states known to influence organ weight and overall health [31,32,33]. Indeed, the histological (H&E) and immunohistochemical (IHC) analyses of the pulmonary tissues from PM_2.5_-exposed mice demonstrated significant elevations in angiogenesis, leukocyte infiltration, and macrophage activation, collectively indicating the development of pulmonary inflammatory responses (Figure 2d). The IHC analysis further confirmed an upregulation of S100A4, a calcium-binding protein associated with cell proliferation, inflammation, and fibrosis [34,35], in the pulmonary tissues of mice exposed to PM_2.5_ (Figure 2d). Furthermore, analysis of bronchoalveolar lavage (BAL) and plasma from the PM_2.5_-exposed mice demonstrated a marked elevation in the levels of S100A4, OPN (osteopontin), and 8-isoprostane (Figure 2e), suggesting the potential for PM_2.5_ exposure to induce oncogenic oxidative stress and inflammatory responses. Additionally, the cell components in BAL exhibited significantly increased levels of neutrophils, lymphocytes, and macrophages, providing further evidence that PM_2.5_ exposure elicits systemic inflammatory responses (Figure 2f). Collectively, these findings suggest that exposure to PM_2.5_ is associated with an increased risk of chronic pulmonary inflammation and oxidative stress, potentially contributing to lung carcinogenesis.

### 3.2. Transcriptomic Profiling

**Figure 3:**
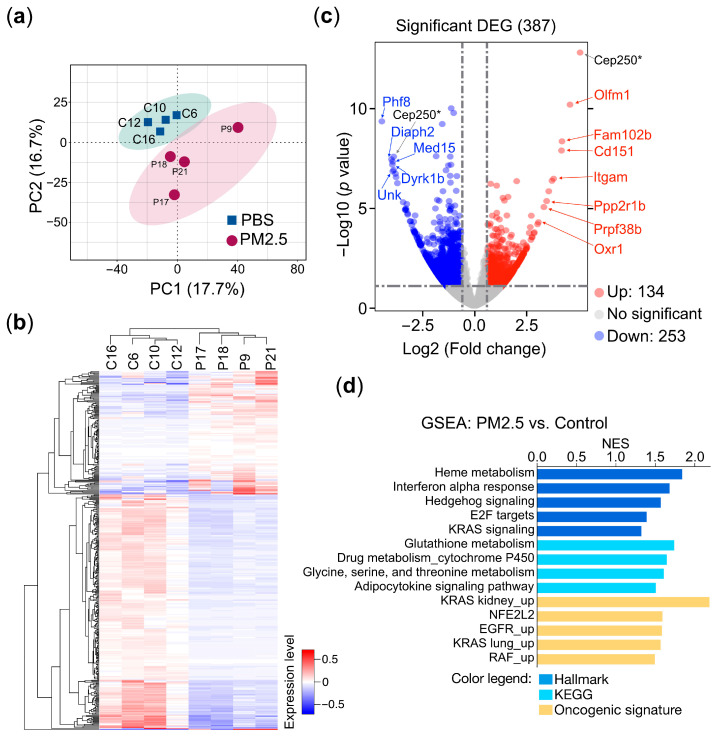
Transcriptomic profiling of PM_2.5_-treated mice. Mice (n = 8) were administered 25 μg of PM_2.5_ samples (n = 4) or physiological saline (n = 4) via oropharyngeal aspiration twice per week and were euthanized 24 weeks after the initial PM_2.5_ treatment. Total RNAs extracted from the mice’s buffy coats were subjected to RNA sequencing (RNA-Seq) analysis. (**a**) Principal component analysis (PCA) of the RNA-Seq data. C, PBS control; P, PM_2.5_-treated mice. (**b**) The heatmap represents the hierarchical clustering analysis. C, PBS control; P, PM_2.5_-treated mice. (**c**) The volcano plot represents the DEGs (differential expressed genes) of PM_2.5_-treated mice compared with the controls. Selected genes are indicated in the plot. (**d**) Gene set enrichment analysis (GSEA). GSEA using the collections from molecular signatures database (MSigDB): Hallmark, KEGG, and oncogenic signature are indicated. The enriched pathways are displayed in a bar plot. The normalized enrichment score (NES) is shown, where |NES| > 1 indicates that a pathway is enriched in the PM_2.5_-treated mice. *p* < 0.05 (*).

To uncover the underlying mechanisms by which PM_2.5_ leads to various pathological conditions and to identify the gene sets specifically associated with PM_2.5_ exposure that could serve as prospective biomarkers, we conducted an unbiased RNA sequencing (RNA-seq) approach to investigate the connections between gene expression and PM_2.5_ exposure. RNA-sequencing-based transcriptional profiling was performed on total RNA samples extracted from the buffy coat cells of mice exposed to PM_2.5_ and the control treatment. The principal component analysis (PCA) clearly distinguished the PM_2.5_ exposure samples from the controls in the two-dimensional PC space (Figure 3a). A heatmap and hierarchical clustering analysis indicated the presence of two distinct clusters of gene expression patterns (group P versus group C) (Figure 3b), suggesting that exposure to PM_2.5_ induced substantial alterations in the transcriptional landscape. Our RNA-sequencing analysis, visualized as a volcano plot, identified 387 differentially expressed genes (DEGs) between the PM_2.5_-exposed and PBS-treated mice. Of these, 134 genes were upregulated, while 253 genes were downregulated (Figure 3c). To further validate the DEGs, we selected the top 12 genes, including both upregulated and downregulated genes, and assessed their specific responses to PM_2.5_ exposure using quantitative reverse transcription PCR (qRT-PCR) in cell line-based experiments. These results are further supported by in vitro cell-based validation (Section 3.3). The gene set enrichment analysis (GSEA) of the DEGs reveals that PM_2.5_ exposure is associated with multiple pathways related to oncogenesis (KRAS and EGFR signaling), redox homeostasis (glutathione and NFE2 signaling), inflammatory response (interferon alpha response), and metabolism (drug metabolism and P450 cytochrome P450 and amino acid metabolism) (Figure 3d), demonstrating the wide-ranging physiological and pathological impacts.

### 3.3. In Vitro Cell-Based Validation

**Figure 4:**
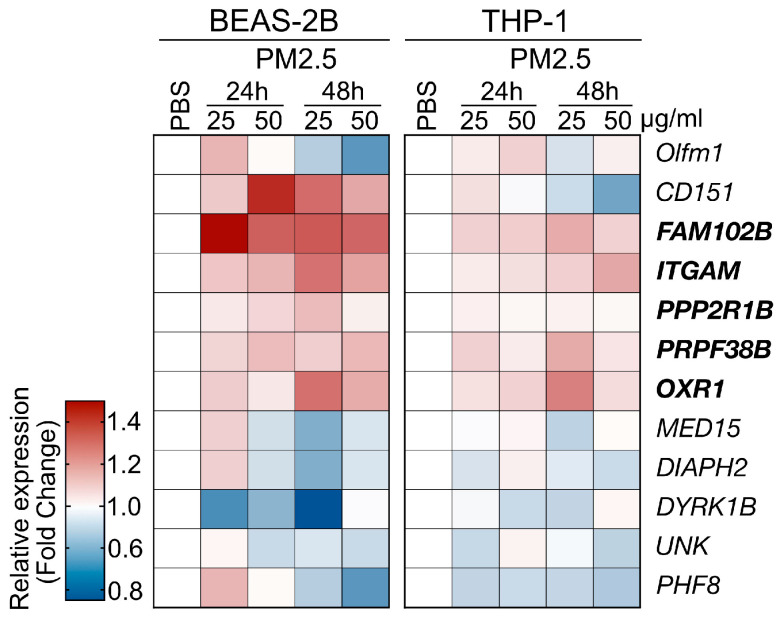
Cell line-based validation for the PM_2.5_-induced genes identified by RNA-Seq analysis. Two human cell lines, BEAS-2B and THP-1 cells, were used to assess the responses to PM_2.5_ treatment. The cells were treated with PM_2.5_ samples at concentrations of 25 μg/mL or 50 μg/mL for 24 h or 48 has indicated. The heatmap depicts the relative gene expression levels, with the tested genes marked.

The PM_2.5_ samples used in the cell line-based validation experiments were collected from the highly polluted Kaohsiung City region in Taiwan, as detailed in the Materials and Methods section. Human THP-1 (human monocyte) and BEAS-2B (human bronchial epithelial cell) cells were exposed to PM_2.5_ at a concentration of 25 μg/mL for 24 and 48 h, after which the levels of gene expression were analyzed using qRT-PCR (primer set listed in Table S1). In this analysis, we selected 12 genes from the RNA-Seq data of a mouse model, which displayed both upregulated and downregulated expression patterns to further investigate the impacts of PM_2.5_ treatment on human cell lines (Figure 4). The validation experiments demonstrated that PM_2.5_ exposure influenced the expression patterns of the selected genes. We observed consistent expression trends for five genes, as observed in the RNA-Seq analysis (Figure 3, marked in bold type). However, while *Cep250* and *Olfm1* were identified as the top two upregulated genes in the RNA-seq, their expression levels lacked consistency across the two cell lines. Consequently, we have chosen the five genes, specifically *FAM102B*, *PPP2R1B*, *OXR1*, *ITGAM*, and *PRPF38B*, for further clinical cohort investigation.

### 3.4. Cohort Study and Biomarker Development

**Figure 5:**
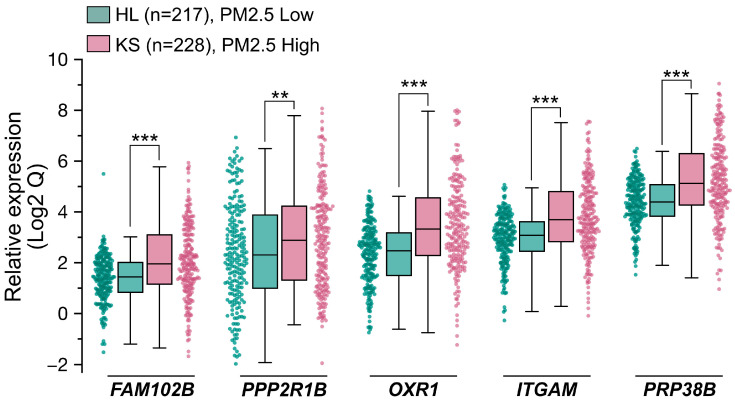
Population cohort investigation for validating five PM_2.5_ responsive genes serving as potential biomarkers. Two distinct population cohorts were recruited, one from Hualien (HL, PM_2.5_ Low, n = 217) City and the other from Kaohsiung (KS, PM_2.5_ High, n = 228) City in Taiwan. All participants were healthy individuals aged 65 or older. cDNA samples were prepared from participants. The expression levels of the 5 genes were quantified with qPCR. Data are presented as box plots and scatter plots showing relative gene expression levels in the HL and KS cohorts. Whiskers extend to the most extreme data points within 1.5 × IQR from the box edges. Comparison between two groups (HL vs. KS) were performed using the Mann–Whitney U Test. Statistical significance is defined as follows: ***, *p* < 0.001; **, *p* < 0.01.

With the goal of identifying predictive biomarkers to PM_2.5_ exposure, we next performed a clinical cohort investigation to assess the feasibility, sensitivity, and specificity of the five genes of PM_2.5_-responsive genes. In Taiwan, two areas, designated as high pollution (KS) and low pollution (HL) for PM_2.5_ exposure, respectively, were selected for this cohort study. The high pollution region (KS), specifically the Xiaogang area, is characterized by high population density and petrochemical industrial activity, while the low pollution (HL) region is known for its low pollution levels and focus on agricultural tourism. This cohort investigation collected blood samples from healthy older adults (ages ≥ 65; for a detailed description, see Materials and Methods) across two geographically distinct cohorts, one from the PM_2.5_ high region KS (n = 228) and one from the PM_2.5_ low region HL (n = 217), and subsequently performed qRT-PCR analysis to evaluate the expression levels (Qs) of the five target genes. The distribution of Q values for the five genes in the KS and HL groups is illustrated in the box plots in Figure 5 and detailed in Table 1. Due to the variability and wide range of Q values across the five genes, Figure 5 presents log_2_-transformed Q values to provide a clearer comparison of their distribution between the KS and HL groups. Across all five genes, the median Q in the KS group were consistently higher than those in the HL group. Notably, the interquartile ranges (IQRs) for the five genes, which represent the variability in Q values, were generally wider in the high exposure KS participants compared to the low exposure HL participants. Additionally, the Mann–Whitney U Test results presented in Table 1 demonstrated *p*-values below the significance level, indicative of statistically significant differences in the examined gene expressions between the two study populations. In summary, the clinical cohort data indicated that the expression levels of five genes were markedly elevated in the KS samples compared to the HL samples. This observation aligns closely with the results from the RNA-Seq analysis and the human cell line validation experiments, implying the potential utility of these genes as predictive biomarkers for PM_2.5_ exposure.

### 3.5. Model Building for PM_2.5_ Exposure Prediction

**Figure 6:**
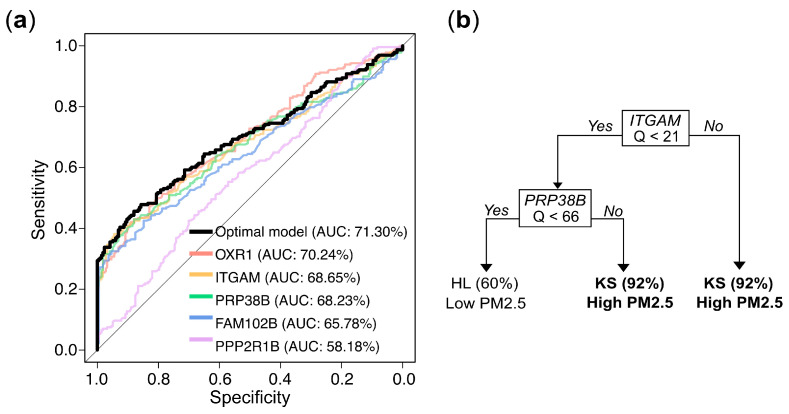
Gene expression levels (Q values)-based prediction of PM_2.5_ exposure: model performance and decision rules. (**a**) Receiver operating characteristic (ROC) curves and corresponding area under the curve (AUC) values for logistic regression models based on individual genes and the optimal multivariate combination. (**b**) Decision rules from a tree constructed via recursive partitioning and pruning, which retained *ITGAM* and *PRPF38B* as the most informative predictors. Samples with *ITGAM* Q ≥ 21 were classified as high exposure (KS) with 92% accuracy. When *ITGAM* Q < 21 and *PRPF38B* Q ≥ 66, the model also predicted high exposure with 92% accuracy. Conversely, when both *ITGAM* Q < 21 and *PRP38B* Q < 66, the predicted probability of belonging to the low exposure group (HL) was 60%.

To further develop predictive models for determining whether an individual belongs to the high or low exposure group based on gene expression values, we used stratified random sampling to allocate 80% of the samples to the training set (n = 356; 182 from KS and 174 from HL) and the remaining 20% to the independent testing set (n = 89; 46 from KS and 43 from HL). The composition of the training and testing sets was consistent with that of the original dataset. The evaluation results for both the training and testing sets are presented in Table 2. The univariate logistic regression models for the markers *OXR1*, *ITGAM*, and *PRP38B* exhibited accuracy rates of approximately 63.20%, 62.92%, and 62.08%, respectively, with corresponding AUCs of 70.24%, 68.65%, and 68.23% in the training set. In the testing set, the accuracy rates were 60.67%, 60.67%, and 62.92%, respectively. These performances slightly outperformed the models constructed using the individual markers *FAM102B* or *PPP2R1B*. The trends in other metrics, including sensitivity, specificity, precision, and F1 score, were similar (Table 2). The optimal multivariate logistic regression model, selected using a bidirectional stepwise procedure, achieved an accuracy of 63.76% and an AUC of 71.03% in the training set, with an accuracy of 61.80% in the testing set, showing a slight improvement over the univariate logistic regression results. The ROC curve and corresponding AUC values for the logistic regression models described above are presented in Figure 6a. The decision tree model demonstrated even better performance, with an accuracy of 70.51% and an AUC of 75.36% in the training set, and an accuracy of 71.91% in the testing set.

The final predictive model, constructed using all available samples, elucidated the predictive mechanism of the markers in distinguishing between high exposure (KS) and low exposure (HL). Univariate logistic regression analyses showed that the odds ratios (ORs) for the five genes in the high exposure group (KS) compared to the low exposure group (HL) were 1.09, 1.08, 1.03, 1.20, and 1.01, respectively, and all were highly statistically significant (Table 3). In the multivariate logistic regression that incorporated all 5 genes and employed a bidirectional stepwise selection approach to identify the optimal combination, only *OXR1* and *PRP38B* were retained. The odds ratios for *OXR1* and *PRP38B* in the KS group compared to the HL group were 1.06 and 1.01, respectively, with both results reaching statistical significance. Nevertheless, in the decision tree analysis, the optimal tree structure, obtained through pruning to prevent overfitting, is shown in Figure 6b. The optimal model included only *ITGAM* and *PRP38B* as predictive markers. The decision tree predicted a high probability (0.92) of belonging to the high exposure group KS when the Q value of ITGAM was over 21. When the Q value of *ITGAM* was below 21 and the Q value of PRP38B was over 66, there was also a 0.92 probability of predicting high exposure. Conversely, when the Q value of *ITGAM* was below 21 and Q value of *PRP38B* was below 66, there was a 0.60 probability of predicting belonging to then low exposure group HL. Notably, the biomarkers selected by the decision tree (*ITGAM* and *PRP38B*) differed slightly from those selected by the logistic regression model (*OXR1* and *PRP38B*), may due to the high correlation (0.82) between *OXR1* and *ITGAM* (Figure S1). While the methodologies of logistic regression and decision trees for modeling predictor–outcome relationships may differ, both analytical approaches yielded reasonable and scientifically valid results.

## 4. Discussion

Using gene expression pattern as biomarkers offers a powerful tool for understanding the biological impacts of PM_2.5_ exposure and their implications for human health. By detecting genetic changes that arise prior to clinical symptoms, these biomarkers provide early warning indicators that can inform public health surveillance and preventive interventions. The evidence that air pollutants can alter gene expression patterns, even at exposure levels below current regulatory thresholds, shows the potential value of biomarker monitoring for protecting public health.

In this study we conducted a comprehensive investigation to identify potential biomarkers of PM_2.5_ exposure. Through a combination of PM_2.5_ exposure mouse models and RNA-sequencing analysis, we were able to pinpoint five specific genes that showed strong association with PM_2.5_ exposure. Importantly, we further validated the relevance of these five genes by conducting both in vitro cell-based experiments with human BEAS-2B and THP-1 cell lines and clinical cohort-based investigations. Building on these findings, we applied machine learning algorithms, including logistic regression and decision tree models, to develop practical predictive models for assessing PM_2.5_ exposure levels in individuals. The models could have important applications in personalized risk assessment and targeted interventions for populations at high risk of PM_2.5_-related health impacts.

Exposure to ambient air pollution, particularly PM_2.5_, has been shown to have significant adverse impacts on cardiopulmonary and metabolic health [1,2,36,37,38,39]. In our PM_2.5_ exposure mouse study, we observed the mice exposed to PM_2.5_ exhibited a significant increase in the mass of vital organs, including the pancreas and lungs (Figure 2c), compared to the control mice. While the precise mechanisms underlying these physiological alternations remain to be further investigated, the findings suggest that PM_2.5_ exposure may induce local or systemic inflammatory responses, which could subsequently lead to increased levels of lung fibrosis and potentially more severe forms of pulmonary sclerosis [40,41]. Further histological examination using H&E staining, as well as the IHC analysis, of the lung tissues from the mice exposed to PM_2.5_ revealed clear evidences of cell proliferation and expressions of markers of oxidative stress supporting the adverse effects of PM_2.5_ on the respiratory system (Figure 2d). Furthermore, we observed that, in the bronchoalveolar lavage (BAL) fluid from PM_2.5_-treated mice, the levels of inflammation and oxidative stress indicators such as osteopontin (OPN), S100A4, and 8-isoprostane were significantly elevated (Figure 2e). OPN is a multi-function protein mainly involved in bone remodeling, wound healing, and inflammation [42,43]. Recent study found that exposure to PM_2.5_ increased the expression of OPN in the lungs of mice [44]. Another study found that OPN levels were higher in the blood of people who lived in areas with high levels of air pollution [45]. S100A4, a protein belonging to the S100 family of small calcium-binding proteins, has been demonstrated to be upregulated in various lung diseases, including lung cancer, pulmonary hypertension, idiopathic pulmonary fibrosis (IPF), and obstructive pulmonary disease (COPD), which led to this protein as a potential serum-based biomarker for predicting various lung-related pathologies [46]. For measuring the level of oxidative stress response induced by PM_2.5_ exposure, the formation of 8-isoprostane can serve as a reliable marker to reflect the level of reactive oxygen species [47,48,49]. This is because when free radicals attack arachidonic acid, a fatty acid found in cell membranes, it triggers a process known as lipid peroxidation and then leads to the formation 8-isoprostane, which can be quantified to assess the extent of oxidative stress induced by PM_2.5_ exposure. Several studies showed that elevated levels of 8-isoprostane are associated with respiratory and cardiovascular diseases [50,51,52].

PM_2.5_ exposure has long been considered to be associated with proinflammatory responses [53]. Accordingly, our BAL fluid analysis showed that PM_2.5_-induced inflammatory response was accompanied by a marked increase in the distribution and number of various immune cells, particularly macrophages and neutrophils, which were dramatically induced in the BAL of PM_2.5_-exposed mice [54]. Notably, He et al. found that the infiltration of neutrophils induced by PM_2.5_ exposure is associated with pulmonary inflammation and the exacerbation of asthma [55]. This suggests that PM_2.5_ exposure can trigger a robust pulmonary immune response, potentially contributing to the detrimental health effects associated with air pollution.

Another adverse effect of PM_2.5_ exposure is the risk of cardiovascular dysfunction, including increased blood pressure, heart rate variability, and the potential development of atherosclerosis and other heart-related complications [56]. In this study, we observed that the exposure to PM_2.5_ had significant impacts on the cardiovascular functions of the mice. The mice exposed to PM_2.5_ exhibited elevated heart rates as well as increased blood pressures, including systolic blood pressure (SBP) and diastolic blood pressure (DBP). This suggests that PM_2.5_ may affect the cardiovascular system through a possible mechanism involving the elevation of oxidative stress and cause inflammation, which can lead to damage of the vessel endothelial cells [57]. This damage to the endothelial cells may result in stiffened arteries, ultimately leading to higher blood pressure and an increased risk of developing coronary artery disease [58]. In addition, PM_2.5_ exposure can also trigger the release of stress hormones, such as cortisol, which can further exacerbate cardiovascular damage and inflammation [59] and lead to a cascade of physiological responses, including elevated blood pressure, increased inflammation, and impaired cardiovascular function, ultimately contributing to the development of various cardiovascular and respiratory pathologies.

Transcriptomic profiling analysis provides an unbiased and comprehensive approach to discover potential biomarkers that can be used to detect or monitor various diseases and health conditions, including the health impacts of PM_2.5_ exposure. Our transcriptomic analysis using PM_2.5_-exposure mouse model with GSEA identified multiple cellular pathways significantly involved in PM_2.5_ exposure. Of these pathways, we noted that oncogenic KRAS signaling, EGFR, and RAF upregulations were significantly enriched in PM_2.5_ exposure, consistent with the widely recognized classification of PM_2.5_ as a carcinogen. This suggests that PM_2.5_ exposure may promote the development and progression of certain types of cancer by activating key oncogenic signaling pathways. Interestingly, pathways involved in immune responses, including the interferon alpha response, were upregulated in the PM_2.5_-treated group. Furthermore, multiple pathways of cellular metabolism and antioxidant signaling, such as heme metabolism, glutathione metabolism, and cytochrome P450 signaling, were highly enriched in the PM_2.5_-treated mice, indicating the key pathways linked to PM_2.5_-associated cellular responses [60,61,62].

Based on the upregulated pathways we identified, we selected five highly upregulated candidate genes for further in vitro and in vivo validation as potential biomarkers. Previous studies have shown that overexpression of the OTX1 gene is associated with the progression of various cancers, including glioma [63], ovarian cancer [64], cervical cancer [65], laryngeal squamous cell carcinoma [66], and esophageal squamous cell carcinoma [67], suggesting that OTX1 may be an oncogenic driver. The ITGAM gene encodes the integrin subunit αM, also referred to as CD11b. This CD11b molecule forms a complex with CD18, creating the integrin alpha-M beta-2 complex (αMβ2). αMβ2 is predominantly expressed on the surface of leukocytes, such as monocytes, granulocytes, macrophages, and natural killer cells, all of which are crucial players in the innate immune response. Mutations or abnormal expression of the ITGAM gene have been linked to certain autoimmune diseases, including systemic lupus erythematosus and asthma. For instance, increased ITGAM levels, which influence macrophage activity, have been associated with neutrophilic asthma. Our study suggests a potential connection between ITGAM overexpression driven by PM_2.5_ exposure and the worsening of respiratory conditions like neutrophilic asthma. As for the PRPF38B (Pre-mRNA Processing Factor 38B) gene, encoding a pre-mRNA splicing factor with RNA binding activity is predicted to interact with the components of spliceosome and thereby facilitate the pre-mRNA splicing process [68]. Previous research has suggested that the PRPF38B gene may function as a prognostic biomarker for breast cancer patients with HER2 overexpression who are receiving trastuzumab therapy [69]. The present study found a positive association between PRPF38B overexpression and PM_2.5_ exposure. Nevertheless, the complex interrelationships between PRPF38B, environmental factors, and health outcomes necessitate further investigation. In our RNA-seq analysis, we also identified two genes, FAM102B (family with sequence similarity 102, member B) and PPP2R1B (protein phosphatase 2 scaffold subunit A beta), as potential novel biomarkers for PM_2.5_ exposure. The precise biological function of FAM102B remains incompletely characterized, while its paralog, FAM102A, has been implicated in estrogen signaling, osteoclast differentiation, and cell membrane trafficking [70,71]. Despite the limited research on the *FAM102B* gene, the lack of a clear association with specific phenotypes or diseases suggests that further research is necessary to fully elucidate the scope of FAM102B’s functions. For *PPP2R1B* elevation, a recent study has demonstrated that the upregulation of the *PPP2R1B* gene, directly targeted by the transcription factor IRF3, is associated with increased dysglycemia and impaired glucose regulation in patients with obesity and non-alcoholic fatty liver disease [72]. We therefore speculate that exposure to PM_2.5_ may contribute to metabolic disturbances in these populations. This study suggests a potential link between the aberrant expression of PPP2R1B caused by PM_2.5_ and the IRF3-mediated inflammatory responses that may contribute to the development of these metabolic disorders. However, the molecular mechanisms underlying the relationship between air pollution exposure and the development of obesity and NAFLD require further investigation.

This study examines the association between the expression patterns of five specific genes (obtained from mouse-based RNA-seq analysis) and distinct cohorts exposed to PM_2.5_. Utilizing data from the cohort investigation, we have developed predictive models based on gene expression profiles to distinguish between populations with high and low exposures. While the results and predictive models presented in this study demonstrate practical utility and potential for clinical application, there are several limitations that warrant further consideration. First, the small sample size and single-country cohort of the data used to develop the models may limit the generalizability of the findings to other populations or settings. In this study, we exclusively used a cohort of healthy elderly individuals to develop the PM_2.5_ exposure model. We speculated that outdoor PM_2.5_ exposure could be more significant for this group than for other susceptible populations, such as mothers and children. The indoor air pollution, on the other hand, could play a more significant role in these other susceptible groups, which may warrant further investigation. Second, the PM_2.5_ samples used in this study were collected from the Taiwan’s KS region, an area renowned for its significant petrochemical and heavy industrial activities, as well as its dense vehicular traffic. Consequently, mass spectrometry analysis of the PM_2.5_ samples has revealed their complex composition, comprising a diversity of organic constituents and inorganic salts. Notably, there is increasing attention on PM_0.1_ particles, which are smaller due to their ability to penetrate deeper into human tissues, where they could potentially exert more pronounced systemic effects. Further investigation is desired to identify specific genetic biomarkers. Third, the lack of individual exposure measures is a common limitation of air pollution epidemiology, because individual-level measures of air pollution exposure are difficult and costly to acquire for a large population. In this study, air pollution data from air quality monitoring stations were used as a proxy for individual exposure, which inevitably led to exposure misclassification. In future studies, integrating individual-level data such as activity patterns and time–activity information with air pollution data would refine exposure assessment and strengthen causal inferences. Additionally, photochemical reaction byproducts, including ozone, nitrogen oxides, volatile organic compounds, and ammonia, have been recognized as significant contributors to airborne pollutants [73,74], even though they are not the central focus of this study. Furthermore, although the cohort investigation in this study utilized five genes as biomarkers, the optimal models derived from both multivariate logistic regression and decision tree analysis included only two markers (*OTX1*/*PRP38B* or *ITGAM*/*PRP38*). This does not imply that the markers excluded from the optimal models are incapable of reflecting high or low air pollution exposure. Rather, it suggests that some markers provided similar information due to high correlations. To avoid overfitting, both statistical and machine learning models tend to include only the variables that provide sufficient and distinct predictive information. The results indicate that each marker has the ability to predict exposure levels, with only minor differences in accuracy. Therefore, in practical applications, the most suitable model can be selected based on the available markers.

## 5. Conclusions

This research presented a multifaceted approach to developing a predictive model for PM_2.5_ exposure, incorporating an animal exposure model, transcriptomic profiling, cell line validation, cohort study, and machine learning model building. Five specific genetic markers, *FAM102B*, *PPP2R1B*, *OXR1*, *ITGAM*, and *PRPF38B*, were identified and a machine learning-derived decision tree was constructed to assess potential PM_2.5_ exposure in individuals.

In particular, from the perspective of computational modeling, we incorporated machine learning algorithms to develop predictive models for assessing PM_2.5_ exposure based on gene expression profiles. By applying both logistic regression and decision tree approaches, we were able to capture complex patterns in the data and generate interpretable classification rules for individual exposure assessment. Notably, the decision tree model provided clear threshold-based decision paths, which may facilitate practical implementation in clinical or public health settings. This application of machine learning highlights the potential of integrating computational modeling with biomarker data to enhance exposure assessment and support personalized environmental health monitoring.

## Figures and Tables

**Table 1:** Summary statistics of gene expression levels (Qs) for the five genes in HL (Low PM_2.5_) and KS (High PM_2.5_) cohorts.

Gene	HL		KS		*p* ^†^
	Median	(IQR)	Median	(IQR)	
*FAM102B*	2.72	(2.27)	3.88	(6.36)	<0.001
*PPP2R1B*	4.96	(12.71)	7.40	(16.30)	<0.01
*OXR1*	5.55	(6.32)	10.07	(18.87)	<0.001
*ITGAM*	8.47	(6.84)	13.01	(20.92)	<0.001
*PRP38B*	20.99	(19.72)	34.93	(59.33)	<0.001

^†^ Mann–Whitney U Test to compare the median between two populations, HL and KS. Statistical significance is defined as *p* < 0.05.

**Table 2:** Comparison of predictive performance for logistic regression and decision tree models on the training set and the testing set.

Metric (%)		Training set						Testing set				
		ACC	SEN	SPE	PRE	F1	AUC *	ACC	SEN	SPE	PRE	F1
Univariate	*FAM102B*	59.55	57.47	61.54	58.82	58.14	65.78	57.30	53.49	60.87	56.10	54.76
	*PPP2R1B*	55.62	52.30	58.79	54.82	53.53	58.18	55.06	53.49	56.52	53.49	53.49
	*OXR1*	63.20	63.79	62.64	62.01	62.89	70.24	60.67	60.47	60.87	59.09	59.77
	*ITGAM*	62.92	63.22	62.64	61.80	62.50	68.65	60.67	60.47	60.87	59.09	59.77
	*PRP38B*	62.08	61.49	62.64	61.14	61.32	68.23	62.92	60.47	65.22	61.90	61.18
Multivariate (Optimal Model)	Logistic Regression	63.76	60.92	66.48	63.47	62.17	71.03	61.80	62.79	60.87	60.00	61.36
	Decision Tree	70.51	90.80	51.10	63.97	75.06	75.36	71.91	88.37	56.52	65.52	75.25

ACC: Accuracy, SEN: Sensitivity, SPE: Specificity, PRE: Precision, F1: F1-Score, AUC: Area under curve. *p* < 0.05 (*)

**Table 3:** Estimated odds ratios (ORs) with 95% confidence intervals (CIs) and *p*-values from logistic regression models assessing the association between gene expression levels (Qs) and PM_2.5_ exposure, comparing high (KS) to low (HL) exposure.

	Gene	OR	[95% CI]	*p* *
Univariate	*FAM102B*	1.20	[1.13, 1.29]	<0.001
	*PPP2R1B*	1.01	[1.00, 1.02]	0.010
	*OXR1*	1.09	[1.06, 1.12]	<0.001
	*ITGAM*	1.08	[1.06, 1.11]	<0.001
	*PRP38B*	1.03	[1.02, 1.04]	<0.001
Multivariate: optimal model	*OXR1*	1.06	[1.03, 1.09]	<0.001
	*PRP38B*	1.01	[1.01, 1.02]	<0.005

* Statistical significance is defined as *p* < 0.05.

## Data Availability

Data are available by contact with the corresponding author. RNA-seq data is submitting to GEO database.

## Supplementary Materials

The following supporting information can be downloaded at https://www.mdpi.com/article/10.3390/toxics13070562/s1. Figure S1: Coefficient of correlation and heatmap between five genes; Table S1: Primer sets used for qRT-PCR; Table S2: The chemical composition of the PM_2.5_ samples used in this investigation was determined by mass spectrometry.
