# Supplementary material for: Population Cohort-Validated PM2.5-Induced Gene Signatures: A Machine Learning Approach to Individual Exposure Prediction
Source: Toxics. 2025 Jun 30;13(7):562. doi: 10.3390/toxics13070562 (PMC12300151; doi:10.3390/toxics13070562)
Supplement: Supplementary file 1 [file toxics-13-00562-s001.zip › toxics-3672750-supplementary.pdf]

Figure S1. Coefficient of correlation and heatmap between 5 genes

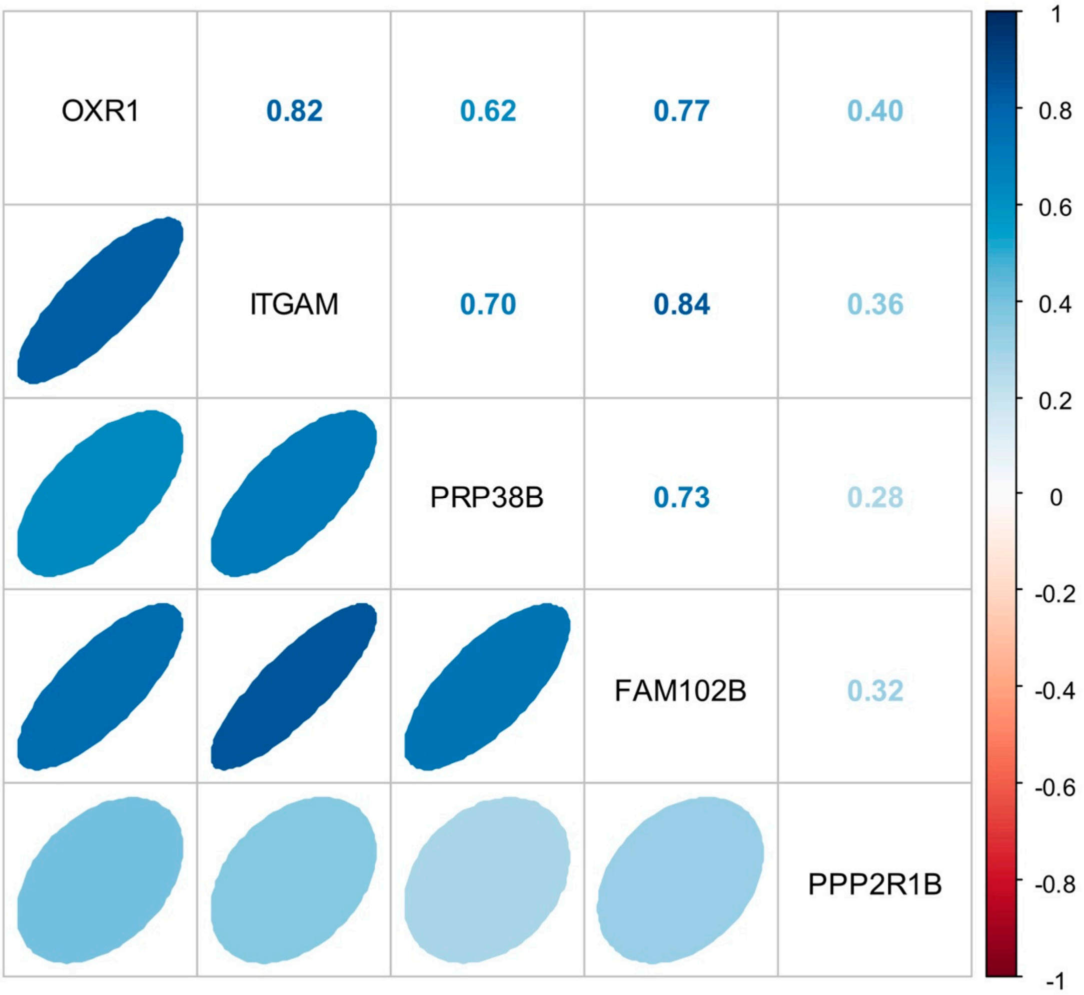

Table S1. Primers used for qRT-PCR	
**Gene**	Sequence (5’ → 3’)
**CE250**	F: AGAGCAGAGCTACAGCGAAT
	R: TCCTTGGCTGCCTGAAGTAA
**CD151**	F: TTCTGCTGGAGATCATCGCT
	R: TGGTGTCCTTCAGGTTCTCC
**FAM102B**	F: TCTCAGCTGAAGCGAGTTGA
	R: GTCCTTCTTCTTCCGCACTG
**ITGAM**	F: AACTTCACGGCCTCAGAGAA
	R: CCCAGGTTGCTGACCTGATA
**PPP2R1B**	F: GCAGCAGTATGAAGGACCAC
	R: AGAGCTCCACAGACAAGGAC
**PRPF38B**	F: GACGTTCAAGGTCTCCAAGG
	R: GGGATCTTCGCCGTTCTTTC
**OXR1**	F: CTGTCCGGAGTTTGAGGTCT
	R: ATCTCCATCAAGCCAAAGCG
**MED15**	F: GGATGCCATGAGGAAAGCTG
	R: AGGCTCTGGAGTGCATTCAT
**DIAPH2**	F: ACGTGAGATGGTTGTCCAGT
	R: TTAACCCAGCTGACCGGATT
**DYRK1B**	F: TTCAGTGGCTCCAATGAGGT
	R: CACCAGGTCCTTCCTGAGTT
**UNK**	F: CCACCTGAGTCAGTCGGAAA
	R: TCCCAGATGCTGCTGTTCAT
**PHF8**	F: GGAGAAGGCTGCTGACATTG
	R: GGAGCTCTCTGACGAACGTA

Table S2. The chemical composition of the PM2.5 samples used in this investigation was determined by mass spectrometry.

Metals (µg/mg of PM)		PAHs* (ng/mg of PM)		Water soluble ions (µg/mg of PM)	
Na	19.119	Napthalene	0.019	Fluoride	0.197
Mg	1.742	Acenapthylene	0.072	Chloride	11.288
Al	4.458	Acenapthene	0.062	Nitrite	2.298
K	10.515	Fluorene	0.027	Bromide	0.645
Ti	0.147	Phenanthrene	0.215	Nitrate	330.162
V	0.287	Anthracene	0.088	Phosphate	0.577
Cr	0.442	Fluoranthene	0.800	Sulfate	213.214
Mn	0.341	Pyrene	0.687	Lithium	0.077
Fe	2.994	Cyclopenta(c,d)pyrene	1.203	Sodium	17.465
Co	0.005	Benzo(a)anthracene	0.689	Ammonium	120.585
Ni	0.106	Chrysene	1.897	Potassium	10.207
Cu	0.174	Benzo(b)fluoranthrene	4.642	Magnesium	1.231
Zn	3.939	Benzo(k)fluoranthrene	1.884	Calcium	6.712
As	0.029	Benzo(e)pyrene	3.854	**Total**	**714.659**
Se	0.045	Benzo(a)pyrene	1.679		
Sr	0.074	Perylene	0.325		
Mo	0.070	Indeno(1,2,3-cd)pyrene	3.614		
Cd	0.008	Dibenz(a,h)anthracene	0.571		
Sb	0.031	Benzo(b)chrysene	0.234		
Ba	3.495	Benzo(g,h,i)peryene	6.134		
La	0.003	Coronene	3.215		
Ce	0.003	Dibenzo(a,e)pyrene	0.247		
Pr	0.000	**Total**	**32.160**		
Nd	0.001				
Sm	0.000				
Yb	0.000				
Lu	0.000				
Pt	0.000				
Pb	0.223				
U	0.000				
**Total**	**48.252**				
